# Inhibitory effects of honey-containing marinades on the production of heterocyclic amines (HCAs) in grilled meat: a review

**DOI:** 10.1007/s13197-026-06686-9

**Published:** 2026-04-28

**Authors:** Shnathini Santharaguru, Anis Syafiqah Yusri, Noraizah Mhd Sarbon, Mohammad Rashedi Ismail-Fitry, Norizah Mhd Sarbon

**Affiliations:** 1 Faculty of Food Science and Agrotechnology, Universiti Malaysia Terengganu, 21030 Kuala Nerus, Terengganu Malaysia; 2 Perdana Square Commercial Centre, Kolej Komuniti Tawau, Batu 4, Jalan Apas, 91000 Tawau, Sabah Malaysia; 3 Department of Food Technology, Faculty of Food Science and Technology, Universiti Putra Malaysia, 43400 UPM Serdang, Selangor Malaysia

**Keywords:** Grilled meat, Heterocyclic amine (HCAs), Marination, Honey

## Abstract

*Grilling* is a dry-heat cooking method that enhances the caramelized flavour of protein-rich foods like lean meats. However, grilling the protein-rich food causes char on the surface, forming heterocyclic amines (HCAs). It was reported that adding antioxidants as a marinating component before cooking inhibited the formation of HCAs. Studies have found that marinades are infused into meat through soaking, enhancing the meat’s flavor, tenderizing the meat fibers, and improving its physicochemical properties. Marinades with Gelam honey, as a primary source of antioxidants, was seen to reduce heterocyclic amine to 50% (Norharm), 5% (Phip) and not detected (IQx) while enhancing flavour with an intense aroma. Hence, this review offers an overview of the HCAs reduction through honey in the marinade and the physicochemical properties of grilled meat. The honey-marinated meat showed higher inhibition of HCAs and better pH properties, moisture loss, cooking loss and colour.

## Introduction

Grilled meat products are known for their smoky and simmered fragrance and flavour (Gisslen 2019). Grilling produces high temperatures in a short, aerobic setting that improves flavour development without causing juice loss, which could harm the texture of the meat. On the other hand, the dry heat method is used in grilling, reducing the cooking time and producing a charred brown colour on the meat product (Ortuño et al. 2021). Moreover, the interaction of reducing sugars with the amino groups of proteins at high temperatures during grilling may lead to the Maillard reaction, which can produce polycyclic aromatic hydrocarbons (Kathuria et al. 2023). Consumers prefer grilled meat as it is comparatively healthy, and the fat is usually melted due to direct heat (Geiker et al. 2021). However, cooking involves dry heat on protein-rich food directed to the surface of the food, causing char on the surface that causes the formation of heterocyclic amine (HCAs).

HCAs are a type of chemical that can cause cancer and mutations that are found in foods heavy in protein, like meat that has been grilled, broiled, or barbecued (Jinap et al. 2015). This organic compound causes a variety of common cancers, like colon, breast, and colorectal, if consumed in high concentrations, as the HCAs damage the deoxyribonucleic acid (DNA) (Kumar et al. 2017). Studies found that a significant reduction in HCAs formation can be achieved through the use of water-soluble vitamins (Wong et al. 2012), Sichuan pepper, Sanchomade extract (Zeng et al. 2018), black pepper (Mousa and Al-Khateeb 2016), and black cumin (Oz 2019) on meat products. Thus, it shows that the marination process significantly reduced the formation of HCAs on grilled protein-rich food products.

A *marinade* is a blend of substances added to raw food, in a paste, powder, or liquid solution. The purpose of marination evolved from enhancing the meat flavour and increasing the yield to improving the meat quality, such as tenderness. It has been observed that when a significant source of antioxidants, like honey and tea, is used as one of the marinating components before cooking, the HCAs concentration in cooked meats is reduced (Shamsudin et al. 2020). In addition, these days, flavor-marinated grilled meat products are popular due to consumers’ growing need for a tasty experience (Nor Hasyimah et al. 2020).

Honey is a sweet, viscous fluid with a pleasant aroma and taste produced by bees from flower nectar or plant secretion. In Malaysia, acacia, gelam, kelulut, and tualang honey are commonly found, and our climate harbours a diverse range of flora and fauna (Sulaiman and Sarbon 2020; Muhammad and Sarbon 2021). Depending on the weather and geographical conditions, honey comprises diverse phenolic, flavonoid, amino acid, ascorbic acid, protein, and carotenoid amounts, as well as antimicrobial and antioxidant characteristics. Honey, as a primary source of antioxidants, was seen to reduce heterocyclic amines in steak. In contrast, the honey in the marinade enhanced the meat’s flavour with an intense aroma by adding nutritional value. The honey-containing marinade has also improved the physicochemical properties of meat products (Shamsudin et al. 2019).

The physicochemical qualities of food are primarily responsible for the product’s final quality. In marinated meat products, a chemical analysis is done on the pH, moisture content, and antioxidant activity. Meanwhile, colour, texture profile, and cooking loss are analyzed regarding physical properties (Nor Hasyimah et al. 2020). Studies conducted on marinated meat showed increased water-holding capacity, cooking loss, and sensory properties while reducing fat retention and total bacteria count. The HCAs formation can be reduced by lowering the pH of marinades through organic acid ingredients compared to their control with no marinade. However, the additional ingredients used to marinate the grilled meat, such as salt and other condiments, also cause changes in the total number of HCAs produced (Nor Hasyimah et al. 2020).

HCAs are synthesized when amino acids in meat juice pyrolyze and occur prominently in grilling and roasting. The HCAs concentration was affected by meat type, temperature, and cooking time. As the cooking technique significantly impacts the quantity of HCAs consumed, it is imperative to provide ongoing updates on HCAs in meat through careful analysis (Nor Hasyimah et al. 2020). However, scientific reviews on the effect of honey-based marinades on the inhibition of HCAs are still limited. Therefore, this review will comprehensively analyze the HCAs reduction through honey in the marinade and the physicochemical properties of grilled meat. Thus, this review aims to critically investigate the effect of honey-containing marinade on inhibiting HCAs formation in grilled meat.

## Grilled meat products

Grilling or pan-frying are widely used as traditional meat-cooking methods, which use high temperatures in an aerobic environment for a short period to maximize flavour development while limiting juice loss that could reduce the quality of meat texture (Ortuño et al. 2021). According to Gisslen (2019), grilled meat products are explicitly known for their smoky or simmered fragrance and flavour. However, preparing food products and cooking are vital in affecting their nutritive value, organoleptic properties, and consumer acceptance.

**Fig. 1:**
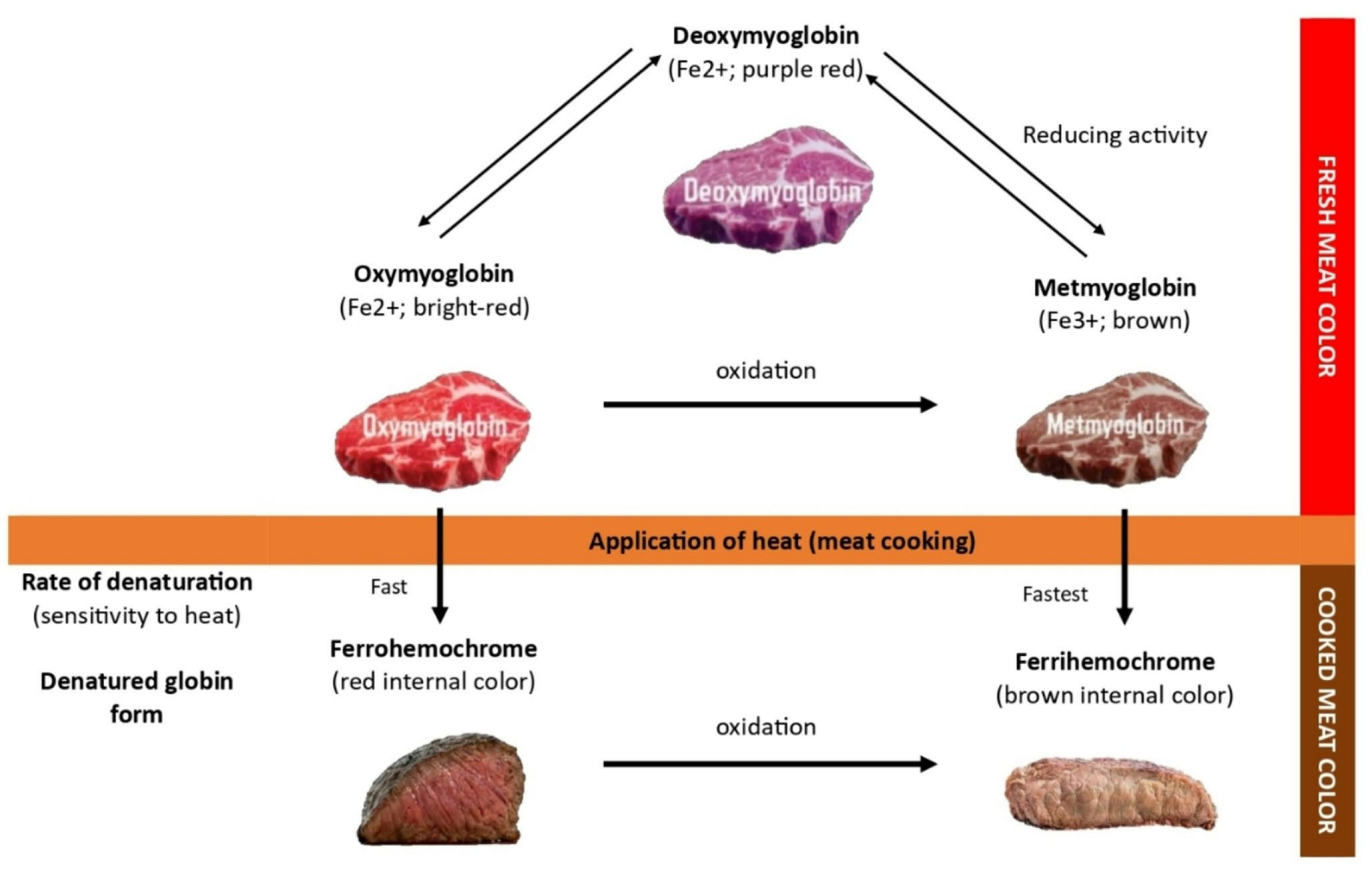
Effects of heat treatment on meat

It was reported that the cooking strategy influences the shading colours, known explicitly as myoglobin, which are identified with warming time and temperature (Ortuño et al. 2021). A study found that myoglobin undergoes denaturation during heat treatment and is converted into metmyoglobin. In the presence of oxygen, myoglobin forms oxymyoglobin (Fig. 1). Thus, a protein-rich product heated at 55 °C undergoes denaturation of oxymyoglobin that causes the colour change. However, the ultimate lean colour, on the other hand, is determined by elements like dry/wet conditions, oxygen availability, and the temperature achieved at the endpoint. It was also observed that dry heat techniques, like grilling, produced a darker surface colour than moist heat techniques because of the dryness and denaturation caused by direct contact with the heating surface (Ortuño et al. 2021). Studies showed that grilled food should be marinated before grilling and cooked to an appropriate degree of doneness. In contrast, after grilling, the surface should be well-browned, charred, moist, and crisp with a moist interior (Gisslen 2019). Entrepreneurs are scrambling to manufacture marinated meat goods because they are becoming increasingly popular in Malaysia. Kenny Rogers Roasters, Mamasab, Pak Mat Western, and several more local product names are actively involved in commercially producing marinated meat.

### Chemistry of grilled meat

#### Heat transfer during grilling

Thermal processing, such as conduction, convection, and radiation, is essential in the food industry, while cooking methods are essential in heat transfer. According to Gisslen (2019), when the food is placed on the grill, it is heated by conduction through the hot grilled meat, while convection takes place when hot air is raised from the burner or charcoal used, and radiation takes place when the glowing burner or coals are used. Conduction occurs when heat or energy is transferred through direct contact, known as heat transmission within a material by molecular agitation, without the material moving. Meanwhile, convection is the process by which heated fluids, such as air or water, are forced to flow away from the source of heat-carrying energy. The fluid’s mass motion then transfers the heat or energy (Kreith et al. 2016).

In addition, the study reported that cooking at higher temperatures resulted in structural changes and protein denaturation due to increased heat flux, which was seen to affect the toughening and tenderization of meat (Suleman et al. 2019). During grilling, roasting, and mechanical stress, the heat mass transfers were rapid because of protein denaturation or contraction, which significantly altered the mass transfer. Flaring of the liquids and fats is released. At the same time, the food is being cooked, and direct contact with the grill rack rods imparts a smoky, mildly burnt flavour to grilled foods. Thus, the product undergoes all three heating mechanisms in the grilling process: conduction, convection, and radiation. Nevertheless, it was observed that the Maillard reaction, characterized by the formation of different substances, was formed during the heating mechanism that occurs in food when it comes in contact with high temperatures (Trevisan et al. 2016).

#### Mechanism of Maillard reaction on grilled meat product

**Fig. 2:**
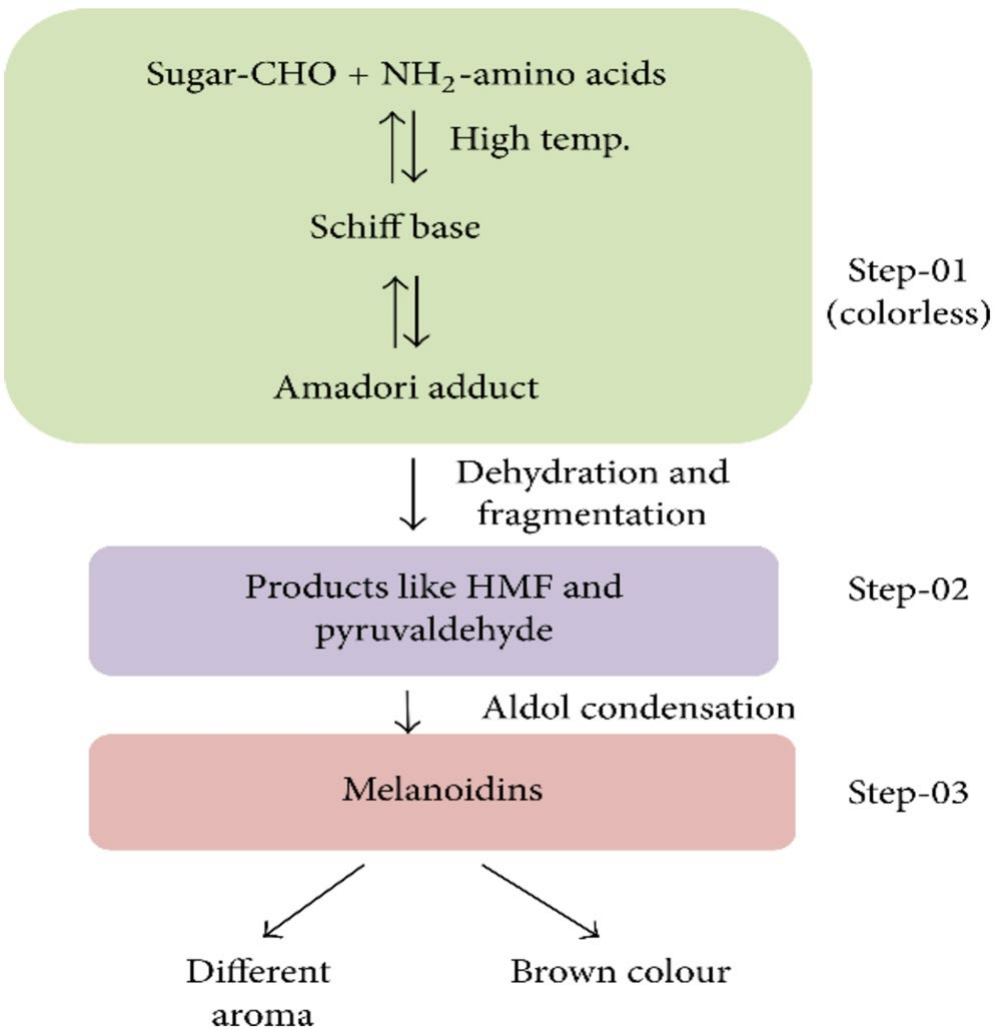
Maillard reaction and flavour production in food

Maillard reaction is non-enzymatic browning that happens because the carbonyl group of reducing sugar and amino acids, peptides, or proteins that could affect the product’s colour, flavour, and stability (Kumar et al. 2017). Figure 2 shows the schematic representation of Maillard reaction products, which consists of three phases. The Maillard reaction combines capacity, pH, water movement, and oxygen, which affect the product’s taste and colour (Berrighi et al. 2020). The grilling method with temperatures over 140 °C encourages Maillard reactions, which form the volatile organic compounds (VOCs), thus responsible for the well-known roast and meaty flavours and the toasted colour of protein-rich meat (Ortuño et al. 2021). Generally, the oxidation of lipids in meat during cooking produces flavour and odour compounds. However, it is also a significant cause of product deterioration, resulting in unpleasant odours, rancidity, texture changes, loss of nutrition, and the development of harmful chemicals (Berrighi et al. 2020). When reducing sugars in honey were heated in acidic environments through the Maillard reaction, an organic product known as 5-hydroxymethylfurfural (HMF) was generated, which had a negative effect as it changed to a non-excretable, genotoxic substance.

### Grilling method

Several grilling methods are widely used, such as charcoal grilling, modified charcoal grilling, electric grilling, infrared-ray grilling, and pan grilling. Although the type of grilling method is essential, the temperature plays a vital role in ensuring the doneness of the grilled product (Park et al. 2017). The grilling temperature chart varies by meat and doneness. Beef steaks are rare at 120–125 °F (49–52 °C), medium at 135–140 °F (57–60 °C), and well-done at 160 °F (71 °C). Pork needs 145 °F (63 °C), and poultry requires 165 °F (74 °C). Thus, it is essential to follow the grilling instructions to ensure the grilled product is safe to consume and does not produce health-deteriorating substances (Kao et al. 2014). A study by Park et al. (2017) found that the grills of pork bellies preheated for 10 min before directly heating food on the charcoal grill, modified-charcoal grill, or infrared-ray grill for 8 min and turned over every 2 min. The grilling process reduces meat’s lipid oxidation, which avoids the deteriorating process that degrades lipid double bonds and synthesizes new molecules, causing off-flavour or lower meat quality (Berrighi et al. 2020). A study conducted showed that the Thiobarbituric acid reactive substance assay (TBARS) value was lowest for grilled foal meat (0.40 ± 0.16 mg MDA/kg) compared to microwave (1.31 ± 0.52 mg MDA/kg), fried (0.41 ± 0.19 mg MDA/kg), and roasted (1.23 ± 0.78 mg MDA/kg) meat (Domínguez et al. 2014).

Although grilled foods are commonly used at home and restaurants, several studies have shown that they pose a significant health risk to the public due to increased levels of carcinogens through the production of benzo[α]pyrene (Farhadian et al. 2011). Compared with charcoal grilling equipment, modified charcoal grilling dramatically reduced the production of benzo[α]pyrene (1.28 g/kg) in the stomachs of roasted pigs. At the same time, infrared grills, electric grills, and grill pans did not affect benzo[α]pyrene (Park et al. 2017). In conclusion, the electric grill, infrared-ray grill, or grill pan is one of the safest grilling methods, ensuring consumers’ safety. However, grilled, smoked, and roasted foods are becoming more popular. Several studies show that these foods pose a greater health risk to consumers due to higher levels of HCAs than foods prepared using simmering, poaching, and steaming (Kao et al. 2014).

## Heterocyclic amine (HCAs)

### Introduction to Heterocyclic amines

Heterocyclic amines (HCAs) are mutagenic and carcinogenic substances with at least one aromatic ring containing a nitrogen atom found in cooked protein-rich foods at the level of parts per billion as a result of the Maillard reaction, with precursors being creatinine, amino acids, and sugars. It was found that the chemicals were generated when amino acids and creatine were combined at high cooking temperatures and were found to be more abundant when meats were overdone or blackened (Yang et al. 2016). The commonly found HCAs in protein-rich products are MeIq (2-Amino-3,4-dimethy-3 H-imidazo[4,5-f]-quinoline), PhIP (2-Amino-1-methyl-6-phenylimidazo [4,5-b] pyridine), DiMeIQx (2-Amino-3,4,8-trimethyl-3 H-imidazo[4,5-f] quinoxaline), and IQ (2-Amino-3 methylimidazo [4,5-f] quinoline under thermic HCAs (Özdestan et al. 2013). Table 1 shows the polarity, compound name, abbreviation, structure, and molecular weight of different types of heterocyclic amine. Previously, the study found that the grill temperature increasing from 180 to 220 °C resulted in a two-fold increase in the 4,8-DiMeIQx content and a nine-fold increase in the 7,8-DiMeIQx content, which affected the physical variable. The rise in 7,8-DiMeIQx concentration was followed by a decrease in tenderness and juiciness when grilled at 220 °C (Buła et al. 2019).

**Table 1:** Polarity properties, compound name, abbreviation, structure and molecular weight of 11 types of Heterocyclic amine (Barzegar et al. 2019)

Polarity	Compound name	Abbreviation	Structure	Molecular weight (g/mol)
Polar	2-Amino-3-methylimidazo[4,5-f]quinolone	IQ		192.2
	2-Amino-3,4-dimethylimidazo[4,5-f]quinolone	MeIQ		212.3
	2-Amino-1-methylimidazo[4,5-f]quinoline	IsoIQ		192.2
	2-Amino-3,4-dimethylimidazo[4,5-f] quinoxaline	4-MeIQx		213.3
	2-Amino-3,4,7,8-tetraimethylimidazo[4,5-f] quinoxaline	TriMeIQx		241.3
	2-Amino-1-methyl-6-phenyl-imidazo[4,5-b] pyridine	PhIP		224.3
Non-polar	1-methyl-9 H-pyrido[4,3-b]indole	Harman		182.2
	9 H-pyrido[4,3-b]indole	Norharman		168.2
	2-Amino-dipyrido[1,2-a:3′2′-d]imidazole	Glu-P-2		184.3
	2-Amino-5-phenylpyridine	Phe-P-1		170.2

### Chemistry formation of hetero-cyclic amine (HCA)

**Fig. 3:**
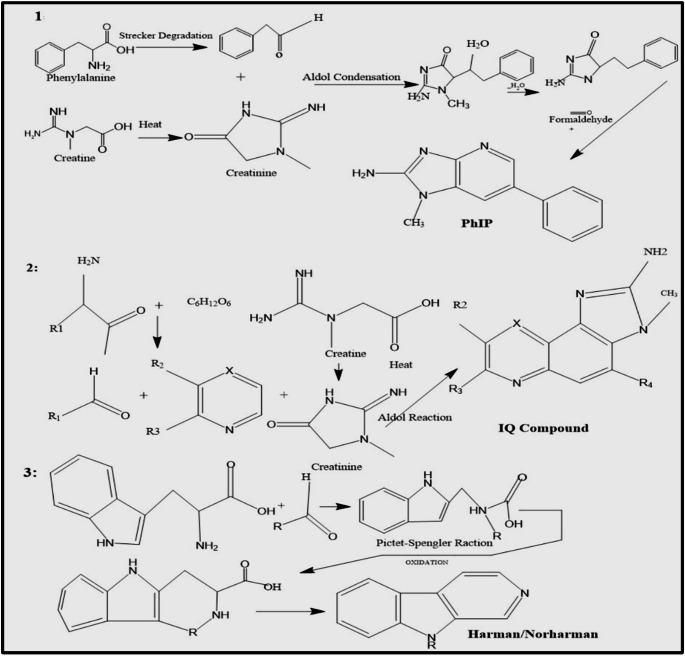
Mechanistics pathway of formation HCA in prevalence in thermally processed meat product

**Fig. 4:**
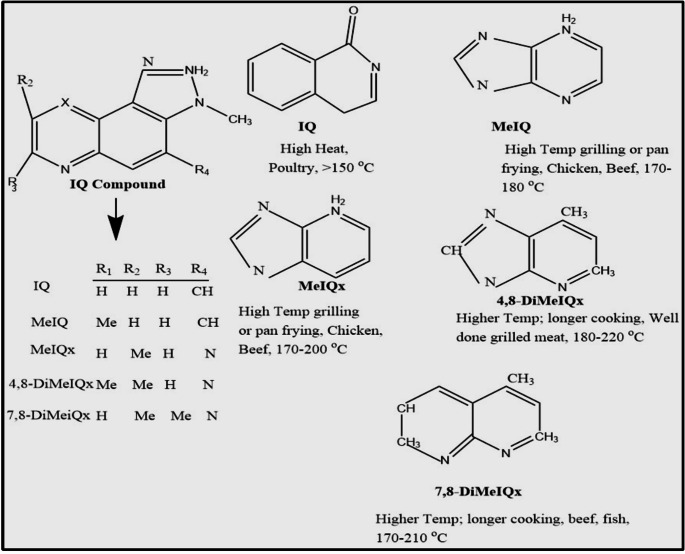
Chemical structure of common IQ-type compound formed after changes in R group at different processing conditions at different meat type

HCAs were classified into two main categories based on their formation processes: amino carbolines and amino imidazo-azoarenes (IQ-type HCAs) (Khan et al. 2022). This classification was determined by distinct pathways through which they were synthesized during cooking, specifically via Maillard reactions or pyrolysis at elevated temperatures (Zamora et al. 2020). Amino imidazo-azoarenes, such as PhIP and IQ-type HCAs, could mainly form through Maillard reaction, which involves amino acids (e.g., phenylalanine), creatine/creatinine and sugars during high-temperature cooking (typically below 300 °C) (Figs. 3 and 4). However, studies showed that PhIP could also form specifically via reaction between phenylalanine and creatine, even without carbohydrates.

PhIP formation is highly influenced by both types of meat and cooking conditions, such as temperature and duration. Its formation is particularly pronounced during high-heat cooking methods like grilling, frying and barbecuing (Hee Pei-Tjun et al. 2024). The process began with the amino acid phenylalanine undergoing Strecker degradation under heat, producing phenylacetaldehyde. Then, the aldehyde reacted with creatinine, yielding intermediate compounds that eventually form PhIP (Hidalgo and Zamora 2022).

In addition, the formation of IQ-type HCAs is another complex process involving several key chemical reactions, which contribute to their mutagenic and carcinogenic potential. As mentioned, primary precursors involved in forming these compounds included creatinine (or creatine), hexoses like glucose, and free amino acids (Zamora et al., 2020). These precursors have undergone various transformations, particularly during the Maillard reaction, involving condensation of amino acids with reducing sugars, forming key intermediates that eventually gave rise to IQ-type HCAs (Hidalgo et al. 2021). Once initial products of the Maillard reaction are formed, such as amino‑carbonyl compounds and Amadori rearrangement products, they further react with other compounds, such as Strecker aldehydes, generated through the degradation of amino acids. This resulted in creating heterocyclic structures such as pyridines and pyrazines (Zamora and Hidalgo 2020).

### Types of heterocyclic amines (HCAs)

Heterocyclic amines (HCAs) were formed during heat, mainly depending on temperature. HCAs are classified into thermic HCAs, such as IQ-type, and pyrolytic HCAs, such as non-IQ-type, which are the two primary types of HCAs based on their high-temperature cooking of protein-rich products (John and Beedanagari 2014). Studies show that in the Maillard reaction through *Strecker degradation*, IQ compounds consisting of pyridine and pyrazine produce hexone and amino acids, respectively (Gibis 2016). *Strecker degradation* is a chemical process in which an amino acid is converted into an aldehyde with a side chain via an amine intermediate. In addition, imidazoquinoline and imidazoquinoxaline are produced when pyridine or pyrazine derivatives react with creatinine, which is created when creatine cyclizes to creatinine during heating. The aldehydes generated alongside creatinine largely synthesize the polar HCA imidazole rings. These rings can then be further classified into polar and nonpolar subclasses according to the polarity of the HCAs (John and Beedanagari 2014).

Radical reactions that occur at temperatures above 300 °C and produce large amounts of reactive fragments are reactions between amino acids and proteins known as pyrolytic reactions that develop non-IQs known as pyrolysis. These fragments are condensed to form new heterocyclic structures, and free-radical reactions produce pyrolytic mutagens of HCAs (Özdestan et al. 2013). Pyrolytic degradation of amino acids is a primary source of pyridine and pyrazines during high-temperature cooking of proteinaceous animal meat (Jinap et al. 2015). For such cooked meats, the burnt flavour is thought to be caused by pyridines, pyrazines, and other volatile chemicals (John and Beedanagari 2014). On the other hand, it was reported that incomplete pyrolysis of organic components like fat, protein, and carbohydrate enhances the formation of polycyclic aromatic hydrocarbons (PAHs) that cause cataracts, jaundice, and skin cancer when exposed (Lu et al. 2018).

### Effect of heterocyclic amine on human health

The peculiar characteristic of heterocyclic amines in human nutrition is their ability to generate Deoxyribonucleic Acid (DNA) adducts and cause mutations when the damaged DNA strands are replicated. These resulted in mutagenic consequences such as chromosomal abnormalities, point mutations, frameshift mutations, and cellular transformations, which altered the genetic information (Lee et al. 2015). These changes eventually manifested as numerous illnesses, such as tumours and cancers of various key organs, posing a serious threat to consumers’ health. Upon consumption, the HCAs’ metabolic activation begins with N-oxidation by cytochrome enzymes, and the acetylation is followed by adduction, mutation, proliferation, and finally malignancy (Rahman et al. 2014).

According to Rahman et al. (2014), the potential carcinogenicity of roasted meat is when combined with finished meat that has other carcinogenic compounds, such as polycyclic aromatic hydrocarbons, N-nitroso compounds, lipid peroxides, and other pro-oxidant compounds, in which it can increase the HCA content besides producing genotoxic compounds. It was seen that by disrupting the DNA, genotoxic substances create mutations that mostly lead to cancer. Thus, HCAs deteriorate human health and cause various types of cancer. In conclusion, the risk of human exposure to HCA is not only influenced by the type of food and cooking method but also by portion size and frequency of consumption. The estimated daily consumption of these chemicals per individual ranged from 0 to 15 g per day. However, it was found that the addition of compounds high in antioxidants, marinades, and lower temperatures reduced the formation of HCAs (Rahman et al. 2014).

## Inhibition of Heterocyclic amine in grilled meat

### Inhibition of heterocyclic amine (HCA)

Reactive carbonyl species (RCS) played a central role in forming HCAs during cooking and were derived from carbohydrates, lipids, and amino acids (Hidalgo and Zamora 2022). These species are highly reactive and contribute to the Maillard reaction and other thermal degradation processes, to generate HCAs. As such, controlling the production of RCS emerged as a promising strategy for reducing HCAs formation in cooked meat products. RCS readily reacted with nucleophiles like amino acids, thiols and antioxidants. Thus, consuming these compounds could influence HCAs synthesis. Any nucleophile capable of interacting with RCS or inhibiting carbonyl-creatinine reaction potentially reduces HCAs formation. For example, thiamine in garlic and onions is identified as an inhibitor of HCAs formation by reacting with RCS, thus limiting HCAs generation (Hidalgo and Zamora 2022).

### Marination

Conventionally, the marination of aquatic products involves the immersion in a liquid solution for 12–48 h. However, this traditional approach often encounters challenges, such as uneven distribution and reduced absorption of marinades in the final product. Moreover, this method is time-intensive, and extensive soaking may result in enzymatic softening, surface bloating and structural damage. In recent years, the assisted marination approach has been widely used in the aquatic industry. The process involves the mechanical movement of the product along with the ingredients in a marination tank, equipped with a vacuum pump. Initially, the water is released with the help of salt through osmosis, facilitating the marinade to be absorbed into the tissue as the muscle structure breaks down (Aberoumand and Aminimehr 2024). Vacuum-assisted marination enhances the penetration of the marinade into the fish flesh by removing air pockets and creating a pressure differential. This leads to a more uniform and improved distribution of the marinade within the flesh, thus, enhancing its quality.

The meat sector has employed marinade technology for centuries. However, the function and perception of marinades have changed throughout time, from flavouring and tenderising meats to increasing yield quality while inhibiting the production of HCAs (O’Neill et al. 2019). This statement was supported by a study showing that adding salt (sodium chloride) reduces cooking water loss, decreases soluble HCA precursors’ transit to the meat surface, and produces fewer HCAs (Neves et al. 2020). A study reported that Sichuan pepper and sansho-amide extract reduced PhIP levels by 89% and 77%, respectively, compared to the control sample of beef patties.

Similarly, IQx, MeIQx, and 4,8-DiMeIQx levels were significantly reduced at 100%, 79%, and 89%, respectively, at the lowest dosage of Sichuan pepper. After applying sanshoamide extract, a sample showed comparable reductions of 100%, 85%, and 89% (Zeng et al. 2018). A study showed that the inhibitory effect on HCA production was correlated to their antioxidant activity, such as honey, with higher antioxidants inhibiting HCAs. Radicals produced during the Maillard reaction were scavenged by antioxidants, causing limitations on the generation of HCAs, verified using electron spin resonance spectroscopy (Gibis and Weiss 2012).

A study also showed that biochemical compounds in spices inhibited the production of HCA (Neves et al. 2020). Table 2 compares different types of marination and the reduction of different types of HCAs. According to a study, marination primarily affects the surfaces of meat products. As a result, the marinade compounds only partially penetrate the interior of meat products, limiting the formation of HCAs and potentially altering customer approval due to overall flavour changes (Gibis and Weiss 2012).

**Table 2:** Inhibition of heterocyclic amine through the marinade

Types of meat	Inhibition method	IQx	MeIQx	4,8-DiMeIQx	PhIP	Norharman	Harman	References
Roasted beef patties	Sichuan pepper	ND	0.33 ± 0.08	0.02 ± 0.00	0.80 ± 0.12	17.96 ± 0.68	2.41 ± 0.16	Zeng et al. (2018)
	Sanshoamides	ND	0.24 ± 0.07	0.02 ± 0.00	1.61 ± 0.21	2.52 ± 0.08	0.50 ± 0.05	
	Chilli pepper	0.26 ± 0.01	0.83 ± 0.01	0.16 ± 0.00	3.74 ± 0.32	2.17 ± 0.19	0.36 ± 0.05	Zeng et al. (2018)
	Capsaicin	0.20 ± 0.03	1.10 ± 0.19	0.09 ± 0.01	0.09 ± 0.01	1.58 ± 0.04	0.28 ± 0.00	
Deep-fried chicken wings	Sugar cane molasses extract	0.86 ± 0.19	0.45 ± 0.03	1.83 ± 0.13	0.03 ± 0.01	1.01 ± 0.18	0.32 ± 0.02	Sabally et al. (2016)
Pan-fried beef sample	Artichoke	1.95 ± 0.02	2.20 ± 0.10	1.19 ± 0.09	7.59 ± 0.59	4.36 ± 0.50	11.72 ± 0.79	Tengilimoglu-Metin et al. (2017)
Fried Beef patty	Ascorbic acid	ND	6.13 ± 0.25	2.12 ± 0.04	6.02 ± 0.13	ND	ND	Wong et al. (2012)
	Niacin	ND	5.99 ± 0.20	2.09 ± 0.09	6.03 ± 0.27	ND	ND	
	Pyridoxamine	ND	4.29 ± 0.49	1.53 ± 0.16	4.24 ± 0.45	ND	ND	
Roast Beef patties	Chilli pepper 0.5%	NQ	0.68 ± 0.05	0.10 ± 0.01	2.60 ± 0.20	0.44 ± 0.02	1.21 ± 0.04	Zeng et al. (2018)
	Chilli pepper 1.0%	0.36 ± 0.02	0.99 ± 0.02	0.21 ± 0.01	3.15 ± 0.09	0.28 ± 0.03	1.49 ± 0.03	
	Chilli pepper 1.5%	0.26 ± 0.01	0.83 ± 0.01	0.16 ± 0.00	3.74 ± 0.32	0.36 ± 0.05	2.17 ± 0.19	
	Capsaicin 2 mg	ND	0.23 ± 0.02	NQ	0.14 ± 0.01	0.27 ± 0.05	1.36 ± 0.03	
	Capsaicin 4 mg	NQ	0.49 ± 0.06	0.05 ± 0.00	0.59 ± 0.06	0.24 ± 0.01	1.80 ± 0.07	
	Capsaicin 6 mg	0.20 ± 0.03	1.10 ± 0.19	0.09 ± 0.01	2.66 ± 0.25	0.28 ± 0.00	1.58 ± 0.04	

### Processing technique

Numerous parameters have influenced the type and amount of HCAs generated in processed food products, including cooking methods, temperature, time, and antioxidant activity. Additionally, research indicates that the most popular home cooking techniques, including frying, roasting, smoking, broiling, and baking, cause the synthesis of HCAs in meat, with each cooking technique producing a unique type of HCA (Ali Khan et al. 2019). However, studies showed that the superheated steam oven processing method produced the lowest level of HCAs, while the traditional oven significantly produced the highest total HCAs. Significantly, the total HCA quantities in sous-vide-cooked samples ranged between 0.036 ng and 0.123 ng, while boiled samples had a total HCA amount of 0.032 ng. Besides, avoiding direct contact with meat surfaces with flames, employing microwave heating as a pre-treatment, and turning meat frequently while cooking were seen to minimize the concentration of carcinogenic chemicals (Rahman et al. 2014).

## Marinade in food preparation

### Introduction of food marination

Marination is applied to meat and meat-based goods to incorporate various nutrients, enhance flavour and juiciness, soften stiff muscles, and increase shelf life. The marinating process impacts meat’s microbiological qualities and sensory benefits (Gargi and Sengun 2021). It incorporates diverse ingredients and additives into the meat using an aqueous or oily solution incorporated into protein-rich meat (González-González et al. 2017). Meat products typically exhibit high water activity and a pH that supports microbial growth; therefore, marination can be employed to reduce the risk of meat-borne diseases (Sengun et al. 2021).

### Marination method

Marination is a standard method of processing used to improve the tenderness, juiciness, taste, and aroma of a diverse range of food products. The process involves soaking or injecting a seasoned solution into food. The solution may increase the shelf-life of marinated food because of its acidic or alkaline nature and specific marinade inputs’ antimicrobial and antioxidant activity (Carnwath et al. 2014). Immersion, injection, tumbling, massaging, and ultrasound-assisted marination manufacture marinated products, intending to increase yield, improve sensory characteristics, and increase tenderness (González-González et al. 2017). Table 3 shows some of the studies related to the type of marination method that affects the meat characterization and degree of marination.

**Table 3:** Impact of marinating techniques on the meat’s characteristics and degree of marination

Marination methods	Holding time	Marinade loss (%)	Marinade absorption (%)	Cooking yield (%)	Cooking loss (%)	Drip loss (%)	References
Immersion	12 h	8.12	–	65.06	34.94	4.48	Gamage et al. (2017)
Injection	12 h	2.29	–	63.91	36.09	2.57	
Tumbling	12 h	2.73	–	71.95	28.10	2.44	
Vacuum-packed marinated (500MPa)	24 h	–	2.9	–	–	–	O’Neill et al. (2019)
Tumbling marination	30 min	–	3.51	32.54	30.00	2.00	U-Chupaj et al. (2017)

Immersion is the oldest method, which entails submerging the meat in the marinade and allowing the ingredients to absorb into the meat over time by diffusion (Vlahova-Vangelova and Dragoev 2014). Meat immersion in acidic marinades causes the solutes in the marinade to be transferred to the food. Injection is where the marinade solution is injected into the meat with needles or probes, and the marinade can permeate the entire piece of meat. Needles or probes are placed into the piece, and the marinade is injected as the probes or needles are withdrawn, spreading the marinade throughout the item. Comparatively, the sensory evaluation results for the injection had a higher preference than the tumbling method. Tumbling or massaging is well-known and approved as a type of physical-mechanical treatment.

Meanwhile, massaging is a gentler method where the meat pieces are rubbed horizontally using paddles. Marination and tumbling or massaging together effectively loosen muscular structures, damage muscle cells, and weaken the connection between myofibers and the meat’s connective tissue. *Vacuum tumbling* is a meat marinating technique used in food processing, supermarkets, and butcher shops to provide a value-added, ready-to-cook product. According to a study, the cooking loss for the vacuum tumbling method was 14.14%, whereas the static marination method had a loss of 23.78% (Gao et al. 2015). However, mechanical tenderizing induces mechanical breakdown, affecting the meat’s texture and appearance.

Several technologies have been recently explored to enhance meat tenderization and speed up mass transfer. An alternative to conventional marinating was ultrasound-assisted marination, or ultrasonic technology. In the fresh and processed meat industries, ultrasonics is becoming increasingly popular as a physical processing technique because of its high penetrating force and convenience. It was regarded as a new method with significant promise for improving and speeding up operations without compromising meat quality, shorter extraction and processing times, lower solvent and energy use, and lower CO_2_ emissions (González-González et al. 2017).

## Honey in the marinade

There are varieties of honey that have been commercialized worldwide. In Europe, there are unifloral honeys, such as black locust, sweet chestnut, lime, sage, and winter sweet honey. Besides, a tropical country such as Malaysia is rich in various types of honey, such as Gelam, Acacia, Borneo, Kelulut, Pineapple, and Tualang honeys. However, the most common types of honey found in Malaysia are Acacia, Kelulut, and Tualang honey (Sulaiman and Sarbon 2020).

Heating of honey is performed at various temperatures and durations from mild temperatures of 50 to 90 °C or 15–120 min, 50–80 °C for 15–60 min, 60–100 °C for 2–20 min and 75 °C to 100 °C for 15 to 90 min. The heat treatment of honey is usually done for two purposes: (a) to alter the tendency of honey to crystallize, and (b) to eliminate the microorganisms that pollute honey (Sulaiman and Sarbon 2020). However, heating honey will change the quality of honey. Heating damage can be shown by evaluating quality control parameters, such as HMF value and diastase activity (Sajid et al. 2022).

### Bioactivity of honey

Honey that consists of hydrogen peroxide (glucose oxide, catalase) and non-peroxide ingredients (lysozyme, phenolic acids, and flavonoids) works as an antibacterial agent. Those compounds give a calming effect when applied to open wounds; it has been used therapeutically in numerous cultures for conditions such as burns, cataracts, ulcers, and wound healing. The mechanism of antimicrobial activity in honey was complex and established by a high concentration of sugar (80% of the weight) and a low pH value, along with the combination of high osmotic pressure, eliminating the osmotolerant microorganism (Gündoğdu et al. 2019). Honey offers a moist wound-healing environment that does not attach to damage tissues due to its physical qualities and high osmolarity. It is intended that the surroundings of the humid wound prevent the colonization of germs.

Honey also has antioxidant attributes because it contains flavonoids, phenolic acids, catalase, peroxides, ascorbic acid, derivatives of carotenoids, and other substances (Muhammad and Sarbon 2021). Antioxidants prevent damage from unstable molecules. However, the composition of phenolic compounds in the honey varies depending on the floral source. Research also demonstrated that stingless bee honey has significantly different physicochemical features, organic acid content, and antioxidant qualities than *Apis mellifera* honey (honeybee honey). Based on the IC_50_ value, *gelam* honey produced by Heterotrigona itama was a more potent source of antioxidants than honey from acacia and starfruit from any of the research species (Sulaiman and Sarbon 2020).

### Effect of heat treatment on honey properties

Heat treatment during honey processing affects honey’s physicochemical, antioxidant, and antibacterial characteristics, with the pH of each honey being less acidic and a darker colour appearing. Heating is done for two primary reasons: to alter honey’s inclination to crystallize and kill contaminating bacteria (Sulaiman and Sarbon 2020). The effect of industrial heat treatment on flavonoids and phenolic compounds has been demonstrated to rely on the specific type of honey being processed (Escriche et al. 2014). Thermal effects on total phenolic compounds and antioxidant activity varied depending on the botanical origin of honey. However, heating changes the quality of the product. Thus, the damage is evaluated through the 5-hydroxymethylfurfural (HMF) value and diastase activity, which are the freshness of honey indicators (Sajid et al. 2022).

## Physicochemical properties and hetero-cyclic amine value of honey-marinade grilled meat

### pH and acidity

pH is the acidity and alkalinity of aqueous media, where most reactions occur in nature, and is determined by the concentration of the hydronium (H_3_O) and hydroxyl (OH) ions. The fall in pH of marinated chicken cubes after marinating was seen to affect the reduction in the percentage of HCA. A study conducted by Jinap et al. (2018) showed that the lowest pH of marinated chicken cubes (5.02%) obtained when tamarind was added in high quantities to honey-containing marinades could be one cause for the high percentage of HCAs reductions in grilled chicken. It has been found that the pH of the marinade influences the water-retaining capacity and water absorption of meat. A low pH marinade, for example, has a better water-holding capacity and absorption due to the increased ionic strength and net negative charge formed in the meat. Lu et al. (2018) stated that lowering the amount of protein-rich meat was seen to increase the cooking loss, which leads to protein denaturation that reduces the water-holding capacity of meat products.

The acidity of honey has been linked to its moisture level. Honey with a high moisture content is more susceptible to fermentation, resulting in high free acidity and low pH values. For example, stingless bee honey samples included more moisture than *Apis* honey. The pH and free acidity values obtained for the stingless bee honey samples ranged between 3.00 and 3.27 and 107.50 to 246.25 meq/kg, respectively, according to the findings of this investigation (Shamsudin et al. 2019). When compared to stingless bee honey, *Apis* honey exhibited much higher pH and lower free acidity values. However, in a study conducted, it was seen that beef satay marinated in *Apis Melifera* honey and *Trigona sp* honey was seen to enhance the fat content compared to the control, where *Apis Melifera* contained 4.75 ± 0.28, *Trigona sp* contained 3.17 ± 0.56, while the control, which was unmarinated, had 1.60 ± 0.20 (Nor Hasyimah et al. 2020).

### Heterocyclic amine quantification

HCAs are another class of unanticipated detrimental culinary toxicants produced by heating (grilling, barbecuing, or broiling) foods high in amino acids and creatinine; they are typically formed in a complicated reaction known as the Maillard reaction, which produces hundreds of reaction products. Thermal treatment decomposes pentoses and hexoses in honey via the non-enzymatic browning reaction, while the Maillard reaction produces undesired volatile products and poisonous chemicals (Lee et al. 2015). The presence of amino acids in honey causes an interaction between the carboxylic group on the reducing end of the sugar molecule and the free amino acids during the Maillard reaction. This is the first step in the synthesis of Amadori compounds with reactive amino (NH_2_) and carbonyl (C_14_O) groups (da Silva et al. 2016). Calamansi was found to be the most efficient organic acid component in reducing HCAs generation in marinades, including table sugar and brown sugar, whereas tamarind was found to be the best organic acid ingredient in marinades containing honey. It was also seen to be the most effective in high concentrations of organic acid. When tamarind, a source of tartaric acid, was added at a high concentration in marinades including honey, the largest percentage of HCAs reductions was obtained. The concentrations of IQx were lowered by 90% (from 5.48 to 1.63 ng/g), MeIQx by 67% (from 8.05 to 1.61 ng/g), IQ by 81% (from 4.09 to 1.26 ng/g), DiMeIQx by 88% (from 10.2 to 4.61 ng/g), MeIQ by 74% (from 4.98 to 2.51 ng/g), and PhIP by 76% (from 9.87 to 4.19 ng/g) (Jinap et al. 2018).

Similarly, the HCA concentrations in grilled beef ranged from undetectable to 344.62 ng/g. Norharman had the greatest concentration in all of the grilled beef samples, ranging from 11.93 ng/g to 314.00 ng/g, followed by harman (3.00 to 14.98 ng/g), MeIQx (not detectable to 5.25 ng/g), AC (0.08 to 2.00 ng/g), and PhIP (0.06 to 1.22 ng/g). The honey-marinated samples included less MeIQx and 4,8-DiMeIQx, which were not found in the unmarinated samples. This observation could be attributed to the lower precursor level of unmarinated samples compared to marinated samples. MeIQx was not found in beef marinated with gelam or starfruit honey; however, it was found in beef marinated with Apis and acacia honey (Shamsudin et al. 2020). This observation could be explained by the various inhibitory effects of the honey-containing marinades. Among the several honey marinades tested, gelam honey had the strongest (95.14 per cent) inhibitory effect on HCAs, which could be attributed to the high antioxidant activity (IC_50_) detected in gelam honey when compared to other honey varieties, as described in our earlier study (Shamsudin et al. 2019).

### Creatine

In the meat industry, cooking loss is a crucial factor that determines the yield, as cooking loss is a mixture of liquid and soluble matter lost from the meat through heat treatment. The heterocyclic pyridine and pyrazine (IQ- and IQx-type) HCAs are formed by the Maillard reaction between hexoses and free amino acids, Strecker degradation of free amino acids, and the transformation of aldehyde and creatine to yield imidazoquinoline and imidazoquinoxaline. As a result, the amounts of HCAs formed in meals are influenced by the precursor amino acids and creatinine (Lee et al. 2015). It’s also responsible for HCAs’ mutagenic activity, and is determined by the Ames test. The Creatinine Colorimetric/Fluorometric Assay kit was used to detect creatinine concentrations enzymatically, and the results were read using an absorbance microplate reader.

A previous study by Nor Hasyimah et al. (2020) evaluated that the unmarinated gas grilled beef had a relatively higher mean of 0.87 ± 0.02, while a reduction was seen in gas grilled beef with *Apis mellifera* honey-spices, which had a value of 0.43 ± 0.03b. which is comparatively lower than gas-grilled beef satay with *Trigona sp*. honey-spices marination, with a value of 0.52 ± 0.01. Comparatively, the creatinine level was seen to be lower in grilled beef meat with a value of 0.04 µmol/g (Shamsudin et al. 2020) (Table 4).

**Table 4:** Quantification of heterocyclic amine after being grilled by honey

Types of meat	Inhibition method	IQx	MeIQx	4,8-DiMeIQx	PhIP	Norharman	Harman	References
Gas-grilled beef satay	Control	0.37 ± 0.10	0.10 ± 0.14	ND	ND	22.68 ± 0.12	1.91 ± 0.0	Nor Hasyimah et al. 2020
	*Apis mellifera* honey-spices marination	ND	ND	ND	ND	2.67 ± 0.45	0.39 ± 0.15	
	*Trigona* Sp. honey-spices marination	0.22 ± 0.07	ND	ND	ND	9.01 ± 0.71	1.30 ± 1.6	
Grilled beef satay	Control	ND	ND	ND	1.22 ± 0.01	141.64 ± 1.43	6.27 ± 0.28	Shamsudin et al. 2020
	Acacia honey	ND	1.78 ± 0.81	3.72 ± 0.67	0.07 ± 0.05	40.60 ± 0.86	3.69 ± 0.02	
	Starfruit honey	ND	ND	4.00 ± 0.95	0.01 ± 0.01	31.55 ± 0.58	3.69 ± 0.02	
	Gelam honey	ND	ND	1.68 ± 0.40	0.06 ± 0.04	11.93 ± 0.27	3.00 ± 0.12	

### Colour

The Maillard reaction mostly influenced the colour of the cooked product; however, a decrease in redness was seen when compared to non-marinated pork. Caramelization, another response, increased the yellowness of cooked pork aged in a mustard-honey marinade. The marinade’s staining effect was also noted by Kim et al. (2014), who confirmed that the colour parameters of raw chicken breasts may be changed by the marinade’s staining effect. The presence of various forms of sugar in the marinated chicken breast samples produced flavour and brown colour, giving it an appealing appearance and taste. The caramelisation of the additional sugars causes the browning, which was induced by the Maillard reaction. Study conducted by Trevisan et al. (2016), mentioned that Grilling to internal temperatures of 80 °C and 100 °C and frying to internal temperatures of 80 °C for home cooking methods suggested by the Food Safety and Inspection Service of the United States Department of Agriculture, and they resulted in meat with low MRP production and a pleasing colour.

### Cooking loss

The cooked meat must reach an internal temperature of at least 70 °C to effectively prevent bacterial growth (Yao et al. 2020). The water loss from the samples after grilling was measured as a percentage of the initial weight. The internal temperatures and weight loss percentages of grilled chicken prepared with three different marinating formulas were compared to those of a control. However, the proportion of weight loss of grilled chicken marinated with honey, 35.2%, was much lower than that of other samples. Honey is a very viscous substance. The honey likely made the marinade more viscous than it would have been with other sugars, which could have affected the weight loss of the grilled chicken. The higher mean concentration of HCAs in the control samples compared to those in the marinated grilled samples could be attributed to the control chicken’s higher percentage of weight loss compared to the marinated grilled chicken (Yao et al. 2020). The lowest cooking loss was most likely caused by caramelization of the sugars in the mustard-honey marinade by coating the surface of the meat, which improved moisture retention.

The cooking loss percentages of the grilled beef samples ranged from 28.10 to 35.38%. Grilled beef cooking loss is due to water loss during the grilling process. Unmarinated samples had a substantially larger cooking loss (35.38%) than other samples. However, the cooking loss of grilled beef marinated with stingless bee honey varied from 28.10 to 28.88%, which was much lower than the cooking loss of unmarinated samples and beef marinated with honeybee honey (Shamsuddin et al. 2020). Unmarinated beef exhibited the maximum cooking loss, which could be attributable to direct heat contact and induced water loss from the flesh.

## Conclusion

In conclusion, this research demonstrates that marinades based on honey can decrease the rate of HCAs synthesis while enhancing the physicochemical characteristics of protein-rich foods. The HCAs concentration might harm the body’s health since it is a carcinogenic food toxicant. Moreover, most research indicates that marinades made with honey considerably reduce the HCAs value, as indicated by the amino carboline (PhIP, Norharman, and Harmon) groups. According to the study’s findings, the most effective method to minimize the creation of HCAs in roast beef throughout the cooking process is to incorporate honey into the marinade mixture. Besides, the chemical composition, such as pH, and physical composition, including cooking loss and colour, of the honey marinated were improved compared to their control. These findings offer a straightforward and helpful method for drastically lowering certain HCAs in grilled meats, which could strengthened with practical implications for the meat industry, food safety regulators, and consumers.

## Data Availability

All data analyzed and used during the current study will be available from the corresponding author upon reasonable request.
